# 非小细胞肺癌患者*K-RAS*基因突变的研究

**DOI:** 10.3779/j.issn.1009-3419.2010.06.007

**Published:** 2010-06-20

**Authors:** 阳 张, 振奎 潘, 星 张, 菲 徐, 力 张

**Affiliations:** 1 510060 广州，华南肿瘤学国家重点实验室 State Key Laboratory of Oncology in South China, Guangzhou 510060, China; 2 510060 广州，中山大学肿瘤防治中心内科 Department of Medical Oncology, Cancer Center, Sun Yet-sen University, Guangzhou 510060, China; 3 266000 青岛，青岛市市立医院肿瘤科 Department of Medical Oncology, Qingdao Municipal Hospital, Qingdao 266000, China; 4 510060 广州，中山大学肿瘤防治中心生物治疗研究中心 Department of Biotherapy Center, Cancer Center, Sun Yet-sen University, Guangzhou 510060, China

**Keywords:** 肺肿瘤, *RAS*基因, 突变, Lung neoplasms, *RAS* gene, Mutation

## Abstract

**背景与目的:**

最近研究显示存在*K-RAS*基因突变的非小细胞肺癌患者难以从辅助化疗中获益，并且对表皮生长因子受体（epidermal growth factor receptor, EGFR）酪氨酸激酶抑制剂（tyrosine kinase Inhibitors, TKIs）耐药。这些发现提示*K-RAS*基因突变情况可作为EGFR TKIs疗效的预测指标。本研究中分析了中山大学肿瘤防治中心非小细胞肺癌患者肺癌组织中*K-RAS*基因突变情况。

**方法:**

收集52例非小细胞肺癌患者的新鲜组织标本，采用PCR技术扩增*K-RAS*基因，然后进行DNA测序并进行相应分析。

**结果:**

在52例患者中，2例患者肿瘤组织中的*K-RAS*基因的12号密码子存在突变（2/52, 3.8%）。统计学分析未发现*K-RAS*基因突变与性别、病理类型、吸烟情况以及肿瘤分化程度和分期间存在相互关系。

**结论:**

非小细胞肺癌患者*K-RAS*基因突变率较低，与亚裔患者相近，而低于白种人患者。

## Abstract (en)

**Background and objective:**

Recent studies indicated that Non-small cell lung cancer (NSCLC) patients with mutant *K-RAS* failed to benefit from adjuvant chemotherapy, and the cancer did not respond to epidermal growth factor receptor (EGFR) tyrosine kinase inhibitors (TKIs). These findings indicated that *K-RAS* gene status can be a biomarker to predict the sensitivity of EGFR TKIs. The aim of this study is to analyze *K-RAS* gene mutations with NSCLC patients in Cancer Center of Sun Yet-sen University.

**Methods:**

52 fresh frozen tumor tissues were collected and *K-RAS* genes were amplified by PCR. Then PCR amplification fragments were sequenced and analyzed.

**Results:**

Somatic mutations in the codon 12 of *K-RAS* gene in tumors were identified from 2 of 52 (3.8%) patients. There were no relationships among *K-RAS* gene mutations and gender, pathology, smoking, differentiation and stage.

**Conclusion:**

The frequency of *K-RAS* gene mutations with NSCLC in our center is very low and is similar to that in Asia patients, and is lower than that in Caucasian population.

目前，表皮生长因子受体(epidermal growth factor receptor, EGFR)酪氨酸激酶抑制剂(tyrosine kinase Inhibitors, TKIs)Gefitinib和Eroltinib已广泛应用于临床，并成为非小细胞肺癌(non-small cell lung cancer, NSCLC)Ⅱ/Ⅲ线的标准治疗。小分子TKIs类药物可通过与ATP竞争结合EGFR酪氨酸激酶域的ATP结合位点而抑制EGFR的活性，从而抑制EGFR下游多条信号通路的活化，从而达到抗肿瘤的作用。

RAS通路是调节EGFR活化下游效应的主要信号转导通路之一，RAS通路的激活对细胞的增殖具有非常重要作用^[1]^。K-RAS属RAS家族的一员，这一家族成员包括K-RAS/N-RAS/H-RAS，它们具有相似的结构与功能。RAS蛋白分子量为21 kDa，又称P21蛋白；它位于细胞膜的内面，可以结合GTP和GDP，具有内在的GTP酶活性。RAS蛋白通常以两种状态存在，活化状态：与GTP结合；失活状态：与GDP结合。在正常细胞内，绝大部分的RAS蛋白以与GDP结合的非活性状态存在。当受到细胞外的刺激时，RAS蛋白与GDP分离，继而与GTP结合而活化，活化后可与相应效应分子相互作用，允许信号的传递，最终这种活化状态因GTP水解为GDP而终止。RAS蛋白由于这种内在GTP酶活性作用，通过将有丝分裂和生长信号从细胞浆传递到细胞核内而参与调节细胞的生长、增殖、分化和调亡等多种生物学过程^[2]^。

在肿瘤细胞中，RAS蛋白可因点突变而丧失内在GTP酶活性，从而不能再阻止刺激细胞增殖信号的传递。15%-20%的非小细胞肺癌患者存在*RAS*基因突变，其中90%为*K-RAS*基因突变；大约有80%的*K-RAS*基因突变发生于12号密码子上，其余则主要位于13号和61号密码子上^[3]^。已发现大约30%的肺腺癌患者存在*K-RAS*基因突变，有研究^[4]^显示*K-RAS*基因突变与吸烟史密切相关，而在终生未吸烟患者中几乎未发现有*K-RAS*基因突变。但亦有研究^[5]^显示不吸烟的NSCLC患者同样可存在*K-RAS*基因突变。此外*K-RAS*基因突变多见于女性患者，而且在早期和局部晚期的非小细胞肺癌中*K-RAS*基因突变与生存期短有密切联系，*K-RAS*基因突变预示预后不良^[6, 7]^。最近的研究^[8]^显示*K-RAS*基因突变与*EGFR*基因突变是相互排斥、独立的。而且*K-RAS*基因突变多发生于应用Gefitinib或Eroltinib治疗过程中疾病进展的NSCLC患者^[9]^。TRIBUTE研究^[10]^提示存在*K-RAS*基因突变的NSCLC患者在应用Eroltinib联合化疗治疗时，其疾病进展时间及中位生存时间均低于单独应用化疗的患者。这些发现提示：*K-RAS*基因突变可能是预测对EGFR TKIs原发耐药的分子标记之一。

目前已知*EGFR*基因突变与EGFR酪氨酸激酶抑制剂Gefitinib和Eroltinib对非小细胞肺癌的有效率密切相关，而且*EGFR*基因突变具有种族差异，亚洲人群的突变率明显高于高加索人群的突变率，*K-RAS*基因突变是否也具有种族差异尚不清楚，目前关于非小细胞肺癌*K-RAS*基因突变国外已有较多报道，而在国内应用基因测序的方法检测肺癌肿瘤组织中*K-RAS*基因突变的研究尚少有报到，我国非小细胞肺癌患者*K-RAS*基因突变的频率、突变特点是否符合国外的报到亦不非常明确，所有这些促使我们做了此项研究。

## 1 材料与方法

### 1.1 标本资料

自2004年7月-2004年10月间共收集了中山大学附属肿瘤防治中心52例可手术的非小细胞肺癌患者的新鲜组织标本，包括肿瘤组织标本和来自同一患者相应的正常肺组织标本。标本自手术过程中获取后于-80 ℃低温冰箱保存。这些患者均未接受过EGFR TKIs治疗。在52例患者中，女性13例(25%)，男性39例(75%)；年龄40岁-76岁，中位年龄为58岁；吸烟患者35例(67.3%)，不吸烟患者17例(32.7%)；病理类型：腺癌27例(51.9%)，鳞癌20例(38.5%)，腺鳞癌5例(9.6%)；Ⅰ期11例(21.2%)，Ⅱ期10例(19.2%)，Ⅲ期25例(48.1%)，Ⅳ期6例(11.5%)；肿瘤分化程度：低分化29例(55.8%)，中、高分化23例(44.2%)，所有病理学类型及TNM分期均按照WHO标准(表 1)。

**1:** 患者特征 Patient characteristics

Characteristics	*n* (%)
Age (year)	
Media	58
Range	40-76
Gender	
Male	39 (75.0)
Female	13(25.0)
Pathology	
ADC	27(51.9)
SCC	20 (38.5)
ADC-SCC	5 (9.6)
Differentiation	
Poorly	29 (55.8)
Well and Moderately	23 (44.2)
Stage	
Ⅰ	11 (21.2)
Ⅱ	10(19.2)
Ⅲ	25(48.1)
Ⅳ	6(11.5)
Smoking	
Yes	35 (67.3)
No	17(32.7)
ADC: adenocarcinoma; SCC: squamous cell carcinoma.	

### 1.2 *K-RAS*基因测序突变分析

首先将新鲜冰冻肿瘤和相应正常组织标本各2 g研磨并用蛋白酶K消化，于55 ℃条件下摇浴15 h，然后应用传统的酚-氯仿法抽提DNA。DNA抽提以后应用PCR扩增*K-RAS*基因的1号和2号外显子(表 2)，其中包含了12、13、61号密码子在内。PCR反应体系为30 μL，包括：2.0 μL DNA(0.2 μg)，3 μL 10×PCR Buffer plus Mg^2+^，1.2 μL dNTP Mixtrue(2.5 mol/L)，2 μL primer(sense+antisense)，0.5 μL TaKaRa Taq(5 U/μL)，21.3 μL DEPC-treated water。反应条件：Exon1初始变性(95 ℃, 5 min)；34个循环：变性(94 ℃, 45 s)，退火(60 ℃, 45 s)，延伸(72 ℃, 45 s)；最终延伸(72 ℃, 10 min)。Exon2初始变性(95 ℃, 5 min)；2个循环：变性(94 ℃, 45 s)，退火(61 ℃, 45 s)，延伸(72 ℃, 45 s)；2个循环：变性(94 ℃, 45 s)，退火(60 ℃, 45 s)，延伸(72 ℃, 45 s)；34个循环：变性(94 ℃, 45 s)，退火(58 ℃, 45 s)，延伸(72 ℃, 45 s)；最终延伸(72 ℃, 10 min)。PCR扩增产物(*K-RAS*基因1-2号外显子)应用DYY-2C型电泳仪及1.5%琼脂糖凝胶电泳检测，有特异扩增片断的PCR产物(图 1，图 2)应用Beckman Coulter CEQ8000 Genetic analysis system和CEQ^TM^ DTCS Quick Start Kit(Beckman Coulter)从正反两个方向进行测序。

**2:** *K-RAS*基因1-2号外显子引物序列 Primer sequences of Exon 1 and exon 2 of *K-RAS* gene

Exon	primer	Primer sequences	Fragment (bp)
1	sense	5'-CGTCTGCAGTCAACTGGAAT-3'	338
	antisense	5'-GAATAATCCTGCACCAGTAA-3'	
2	sense	5'-GGTGCTTAGTGGCCATTTGT-3'	426
	antisense	5'-TGCAATGGCATTAGCAAAGAC -3	

**1:**
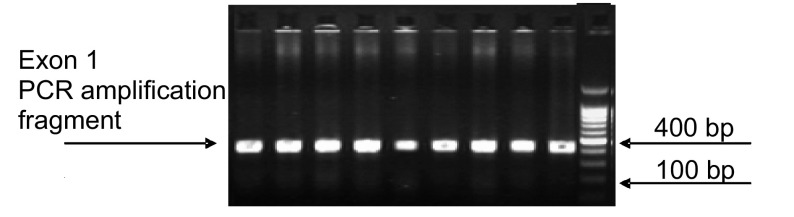
*K-RAS*基因1号外显子PCR扩增产物凝胶电泳成像 Gel electrophrosis photographs of PCR amplified products for *K-RAS* codon 1

**2:**
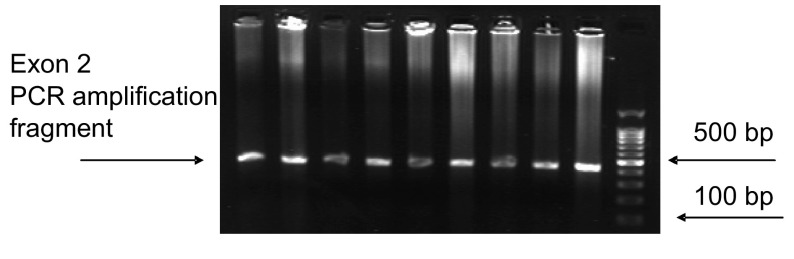
*K-RAS*基因2号外显子PCR扩增产物凝胶电泳成像 Gel electrophrosis photographs of PCR amplified products for *K-RAS* codon 2

### 1.3 统计学分析

应用*Fisher's*检验分析了*K-RAS*基因突变与性别、组织学类型和吸烟状态、分化程度及分期之间的关系，以*P* < 0.05为有统计学差异。

## 2 结果

### 2.1 *K-RAS*基因12、13、61号密码子的突变情况

在52例患者中，2例患者肿瘤组织中的*K-RAS*基因的12号密码子存在突变(2/52, 3.8%)。而相应正常组织中均未检测到有突变的存在。突变类型均为单个核苷酸的替代突变G→T(G12C)和G→A(G12D)(表 3，图 3，图 4)。

**3:** 非小细胞肺癌患者*K-RAS*基因突变情况 *K-RAS* gene mutations with NSCLC patients

Patient	Sex	Histotype	Smoking	Exon (codon)	Nucleotid	Amino Acid
12	Female	ADC	N	1(12)	G→T	G12C
78	Male	SCC	Y	1 (12)	G→A	G12D

**3:**
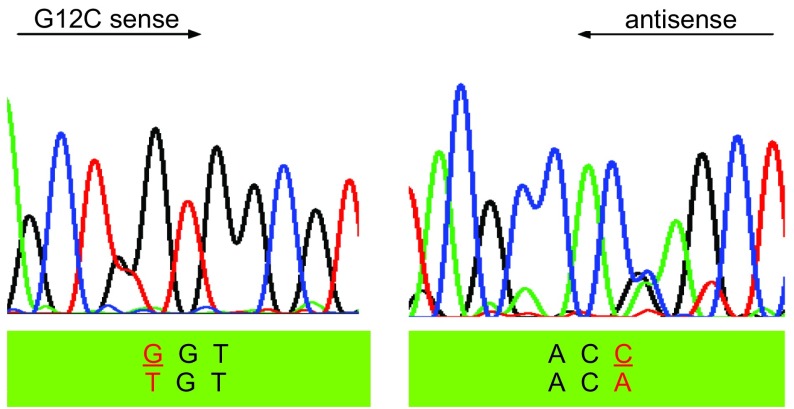
*K-RAS*基因12号密码子G→T突变 G→T mutation in the codon 12 of *K-RAS* gene

**4:**
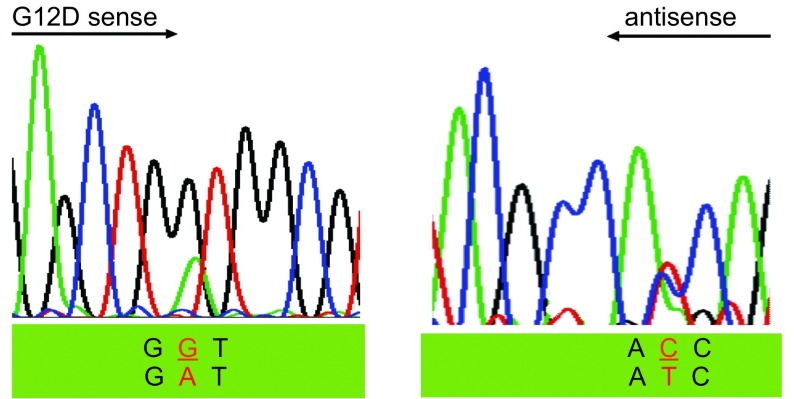
*K-RAS*基因12号密码子G→C突变 G→C mutation in the codon 12 of *K-RAS* gene

### 2.2 *K-RAS*基因突变同患者临床特点之间的关系

在2例发生*K-RAS*基因突变的患者中，1例为腺癌(1/27; 3.70%)，1例为鳞癌(1/20; 5.00%)；1例为女性(1/13; 7.69%)，1例为男性(1/39; 2.56%)；1例为吸烟者(1/35; 2.86%)，1例为非吸烟者(1/17; 5.88%)。*K-RAS*基因突变同性别、病理类型、吸烟情况以及肿瘤分化程度和分期均无相关性(表 4)。

**4:** *K-RAS*基因突变同患者临床特点之间的关系 Relationships between *K-RAS* gene mutations and patient characteristics

		K-RAS mutation					
		mutation (*n*=2)			No mutation (*n*=50)		
	*n*	*n*	%		*n*	%	*P*
Gender							
Male	39	1	2.56		38	97.44	1.000
Female	13	1	7.69		12	92.31	
Smoking							
No	17	1	5.88		16	94.12	1.000
Yes	35	1	2.86		34	97.14	
Differentiation							
Poorly	29	1	3.45		28	96.55	1.000
Well and moderatly	23	1	4.35		22	95.65	
Histologic type							
ADC	27	1	3.70		26	96.30	1.000
SCC	20	1	5.00		19	95.00	
ADC-SCC	5	0	0.00		5	100.00	
Stage							
Ⅰ	11	1	9.09		10	90.91	0.774
Ⅱ	10	0	0.00		10	100.00	
Ⅲ	25	1	4.00		24	96.00	
Ⅳ	6	0	0.00		6	100.00	

## 3 讨论

虽然EGFR酪氨酸激酶域突变才发现不久，但其已经成为临床判断NSCLC患者对EGFR酪氨酸激酶抑制剂Gefitinib和Eroltinib治疗有效的指标。尽管非小细胞肺癌*K-RAS*基因突变发现已超过20多年，但似乎最近其临床价值才引起关注。而且最近的研究^[11, 12]^显示存在*K-RAS*基因突变的非小细胞肺癌患者难以从辅助化疗中获益，并且对EGFR酪氨酸激酶抑制剂耐药^[9]^。这些提示*K-RAS*基因突变情况可作为EGFR酪氨酸激酶抑制剂疗效的预测指标。在我们的研究中仅发现2例患者存在*K-RAS*基因突变(2/52; 3.8%)。腺癌的突变率为3.70%(1/27)，鳞癌的突变率为5.00%(1/20)；这一点与先前报道的白种人患者腺癌*K-RAS*基因突变率为30%明显不相符合^[6]^，但却与近来来自韩国(8.7%)、日本(13%)和香港(9.8%)等学者亚裔患者肺腺癌K-RAS基因突变的报道相近^[9, 13, 14]^。造成这种明显不同的原因还不清楚，我们推测存在差异的原因很可能与种族相关，对此尚需要进一步研究证实。因为亚裔患者*K-RAS*基因的突变率太低，因此*K-RAS*基因突变情况作为EGFR酪氨酸激酶抑制剂疗效的预测指标在亚裔人种中可能并不适用。在2例突变的患者中1例为吸烟者(1/35; 2.86%)，1例为非吸烟者(1/17; 5.88%)，统计分析显示*K-RAS*基因突变与吸烟情况无相关性。这与部分研究认为*K-RAS*基因突变与吸烟史密切相关结果不一致^[4]^，但与部分研究*K-RAS*基因突变也可见于吸烟者的观点一致^[5]^。尽管大量研究分析试图将吸烟史与K-RAS基因突变联系在一起，但缺乏患者吸烟史的详细资料，并且多数报道仅研究了较少数量的不吸烟者。另外我们知道，尽管结肠癌患者有较高的*K-RSA*基因突变情况，但是结肠癌并不与吸烟相关。此外，单因素统计分析显示*K-RAS*基因突变与性别、病理类型、肿瘤分化程度以及病理分期之间均无相关性(表 4)。先前的数据显示大约有80%的*K-RAS*基因突变发生于12号密码子上，其余则主要位于13号和61号密码子上，突变类型主要为单个核苷酸替代突变^[4]^。在我们的研究中2例患者肿瘤组织中的*K-RAS*基因突变均发生于12号密码子，突变类型单个核苷酸的替代突变G→T(G12C)和G→A(G12D)，13和61号密码子未发现有突变的存在。这与先前的报道也是相符的。我们的研究结果既有与先前的研究一致的地方，也有不同的地方，鉴于我们病例数量较少，对此尚需要进一步扩大病例数进行研究。

## 4 结论

我中心非小细胞肺癌患者*K-RAS*基因突变率较低，与日本、韩国等亚裔患者*K-RAS*基因突变率相近，而低于白种人患者的*K-RAS*基因突变率。*K-RAS*基因突变主要为12号密码子上的单个核苷酸的替代突变。
